# Empirical study of employee loyalty and satisfaction in the mining industry using structural equation modeling

**DOI:** 10.1038/s41598-022-05182-2

**Published:** 2022-01-21

**Authors:** Shoukun Chen, Kaili Xu, Xiwen Yao

**Affiliations:** Key Laboratory of Ministry of Education on Safe Mining of Deep Metal Mines, School of Resources and Civil Engineering, Northeastern University, Shenyang, 110819 China

**Keywords:** Human behaviour, Risk factors, Psychology and behaviour

## Abstract

Mining is a high-risk industry and a crucial economic driver that has a crucial role in the economies of countries worldwide. The implications of the labor market on the sustainability of the mining industry have increased the importance of sustainable human resource management at the strategic level of mining and safety management. In this article, from the perspective of management research in an energy production enterprise, we investigated the relationship between employee loyalty and employee satisfaction through a survey that targets employee loyalty, work quality, and job satisfaction and the relationship between enterprise image and switching costs. Based on service profit chain theory, we established a research model for mining employee loyalty, and 500 miners in a typical extreme mining environment in China were surveyed. The study hypotheses were tested using a structural equation model and an employee loyalty model, followed by empirical testing of the models. Employee loyalty was significantly associated with enterprise image and employee satisfaction, work quality indirectly affected loyalty through satisfaction, and the impact of switching costs on employee loyalty was not significant. We provide strong empirical evidence to help enterprises improve sustainable human resource management and regulatory policies, with important implications for safety production. Our study also provides a useful reference for further studies of sustainable human resource management in mining.

## Introduction

Mining is a high-risk industry that plays a crucial role in the economy in countries around the world^1^. Despite extensive efforts to improve mine safety, accidents still pose a threat to the sustainability of the mining industry, as they can lead to the death and injury of workers, property degradation and environmental damage^2^. According to statistics^3–5^, the main causes of accidents are violation of operating rules or labor discipline and poor production environment. Ninety-seven percent of mine accidents are caused by miners' unsafe behaviors^6,7^.

With the deepening of the related research, multi-perspective analysis and research on unsafe behaviors and safety management have been carried out in many industries. Human error is defined as the failure of planned actions to achieve the expected goals, although human error is a major cause of unsafe behavior in accidents^8–10^. Since people's attitudes are always reflected in their behaviors, behaviors are most likely determined by attitudes. The relationship among a contractor's risk perception, attitude and behavior has been analyzed^11^. Zohar and Luria^12^ believed that the security climate was a subset of organizational climate, and employees' behaviors were shaped by their expectations of organizational value and the reward system. Mohamed^13^ used a structural equation model (SEM) to study the relationship between safe climate and safe work in a construction site environment and proved that safe work behavior is the result of a safe climate. The social communication between workers and managers on organizational safety is considered important^14^. In discussing social exchange theory, it is believed that when one party behaves in a way that provides benefits to another party, an implied obligation for future reciprocity is created. Therefore, psychological contract theory is considered the result of social exchange theory, which can be considered in exploring the relationship and different motivations between managers and workers in terms of safety^15^. Intrinsic characteristics can strengthen the loyalty relationship between workers and managers^16^. It was found that employee loyalty was positively correlated with labor productivity^17^. They thus proposed management policies and countermeasures to improve employees' loyalty to the organization. When organizations fail to meet individual expectations, the employee turnover rate in the construction industry will increase, which will lead to the collapse of the employment relationship and thus facilitate accidents^18^. Therefore, construction units should establish the understanding and management mechanism of employees' psychological contracts and develop employee incentive schemes to enhance employees' loyalty to the organization. Some studies have confirmed the mediating effect between loyalty factors and satisfaction. Othman^19^ quantified the drivers of customer loyalty and satisfaction through an international questionnaire and took satisfaction as an intermediary variable to express employee loyalty. In high-risk industries, such as mining, previous studies have focused on unsafe employee behavior, accident causes and the relationship between accidents and people's behaviors, but very few studies have paid attention to the interaction between employee satisfaction and loyalty in relation to accidents. Therefore, this study mainly investigated the interrelationship among employee image, switching cost, work quality (comfort, reliability, responsiveness and empathy), employee satisfaction and loyalty in the high-risk mining industry and establishes a loyalty conceptual model. It provides new ideas and theoretical support for safety management decisions and dynamic management in mining enterprises.

However, sustainable human resource management (HRM) can help mining enterprises establish an attractive enterprise brand that can address the different needs and expectations of potential and existing employees without damaging long-term corporate image, thus promoting sustained competitive advantage^20^. Sustainable HRM can help mining enterprises attract and retain high-quality employees, improve the management of employees and reduce unsafe behaviors to ensure production safety^20–22^. Therefore, it is urgent to study employee satisfaction and loyalty as important factors of sustainable HRM.

In this study, we investigated employee satisfaction and loyalty in the largest copper mine in China (Pulang Copper Mine). These interviewees are suitable for this study due to their strong industry representation. The main reasons are as follows. First, this mine is the largest copper mine production enterprise in China, and a series of face-to-face conversations with managers and miners indicate that there are a large number of miners in this mine with high mobility. Second, our conversation with the miners before the questionnaire survey showed that they were concerned about the safety management of the mine and had a good understanding of the safety production issues. A literature review revealed that research on the loyalty of miners in plateau mines is lacking, so this study comprises an exploratory study. Regarding the framework of this model, previous studies applied employee satisfaction as a single component to investigate the mediating effect of employee loyalty, while this study considered satisfaction a key component according to the characteristics of mine employees to identify specific relationships of employee loyalty and to describe how these relationships affect employee loyalty. This study closes the research gap and contributes important knowledge to current research on the loyalty of miners and mine safety management in plateau mines. However, the intention of this paper is to examine the psychological changes and influencing factors of job satisfaction and loyalty of mining employees in high-altitude and working conditions in environmentally challenging environments.

The research framework of this paper is described as follows: First, we reviewed the relevant literature and defined the concepts of employee satisfaction and loyalty to provide a theoretical basis for the study. The influencing factors of employee satisfaction and the relationship between these factors and employee satisfaction and loyalty were described, as was the relationship between employee satisfaction and employee loyalty. Second, on the basis of previous studies, the concept of the employee loyalty hypothesis model is proposed. The SEM based on variables and Amos software were used to test the hypotheses. Then, the results were evaluated, discussed and summarized. Finally, the research implications, limitations and directions for future research were presented in the conclusion.

## Literature review and hypotheses

### Employee satisfaction

Employee satisfaction is defined as an index of preference for experienced work, while preference for external opportunities depends on the information available at a given time^23^. Employee satisfaction also includes a comparison of future expectations of one's own work and external future opportunities^24^. Employee satisfaction is considered to be the overall feeling about a job or the attitude towards the job^25^. Rice et al.^26^ proposed that satisfaction was to some extent determined by the differences generated in the process of psychological comparison, which involved the assessment of current work experience according to an individual’s comparison criteria. Employee satisfaction is defined as the pleasant or positive emotional state generated by the evaluation of a job or work experience^27^.

However, employee satisfaction is an extremely important variable that can reflect the general mood and thinking of employees about the nature of work and the working environment and conditions. Therefore, employee satisfaction refers to employees' expectations of the workplace and attitude towards work and psychologically determines their work behavior ability and risk perception. Thus, job satisfaction is a function of one's needs being met to some extent^28^.

Scholars have put forward many theories about the definition of employee job satisfaction. For example, motivation-hygiene theory states that the factors that create job satisfaction are separate from those that lead to job dissatisfaction^29^. Factors that lead to job satisfaction are called motivators and include achievement, recognition, the job itself, responsibility and promotion. The factors that prevent job satisfaction and lead to job dissatisfaction are called hygiene factors and include administrative policies, supervision, remuneration, interpersonal relationships and working conditions and quality^30^. Moreover, the impact of employee satisfaction has been analyzed considering five factors, namely, empowerment and participation, working conditions, rewards and recognition, teamwork and training, and personal development^31^.

### Employee loyalty

Employee loyalty refers to employees having deep feelings for the enterprise, being willing to collectively grow with the enterprise, having a sense of responsibility and mission in work, contributing their intelligence and wisdom to achieve the enterprise's goals, and fulfilling their role in helping the enterprise to achieve its strategic goals^32^.

Zhao and Li^33^ proposed that employee loyalty refers to employees' recognition of the enterprise and their attitude and behavior of performing their best, which is embodied in their consistency with enterprise values and policies in ideology.

Employee loyalty refers to the degree of employee loyalty to the enterprise, which is a quantitative concept. Enterprise loyalty means that the enterprise creates an acceptable corporate culture and environment for its employees and provides them with development opportunities and material rewards, so that they can devote themselves wholeheartedly to their work and integrate their personal development into the development of the enterprise^34^. Since employee loyalty and enterprise loyalty have an interactive relationship, employee loyalty originates from the enterprise loyalty to employees^34^. Therefore, loyalty is characterized by a strong desire to remain a member of the organization, which plays a positive role in retaining members of the organization.

All the above researchers acknowledge that employee loyalty plays an important role in enterprise development. In general, loyalty mainly includes behavioral loyalty theory, attitudinal loyalty theory and synthesis theory. This paper adopts the abovementioned theories to investigate the loyalty of miners in the mining industry. Especially in the plateau mine, the environment is poor and the working conditions are more complex, which causes the loss of a large number of miners and technicians and creates severe challenges to production safety management. The paper attempts to research the influencing factors of miners' loyalty. This research is helpful for plateau mine managers who wish to master the psychological contract of miners, which is critical to mine management decision-making and sustainable human resource management.

### The influencing factors of employee satisfaction and loyalty

In 1994, a service management research group composed of James L. Heskett and W. Earl Sasser. Jr and other professors of Harvard Business School proposed the "service profit chain" model. Service profit chain theory holds that there is a direct correlation between employee satisfaction, employee loyalty, employee work quality and efficiency, customer value, customer satisfaction, customer loyalty, and enterprise profit and growth. According to the theory and the special situation of the mining industry, this paper constructs the research concept hypothesis model.

#### Employee work quality

Employee work quality drives employee satisfaction. The factors that make employees satisfied with an enterprise generally include two aspects: first, the external work quality provided by the enterprise, such as salary, welfare, comfortable working environment and other external conditions that can be seen; second, the quality of internal work provided by the enterprise, including the selection and development of employees, reward and recognition, information access channels and work design, organizational leadership and other aspects.

Employee work quality refers to the employee's evaluation of the overall superiority of working conditions and psychological contract satisfaction, which is a cognitive quality^35^. This study investigates four aspects of work quality (comfortability, reliability, responsiveness and empathy). Most studies indicate that employee satisfaction is mostly based on work quality. Host and Knie-anderson^36^ noted that reliability and certainty can predict satisfaction in five aspects of job quality. However, Parasuraman et al.^37^ believed that improving work quality can help build employee loyalty, and work quality has a positive impact on employee loyalty. Although the above studies support the positive relationship between job quality and employee loyalty, most studies have found that the direct relationship is not significant^38,39^ because the relationship between work quality and employee loyalty is affected by the mediating variable of employee satisfaction. Therefore, the following hypothesis is drawn in this study.

##### Hypothesis 1 (H1)

Work quality has a positive effect on employee satisfaction.

## Enterprise image

Enterprise image refers to the overall impression of the enterprise among employees. The enterprise image exists in the hearts of employees and drives their intention of employment in the enterprise. The formation of enterprise image is the visibility of the enterprise to society and the subjective overall evaluation of the internal management and production conditions in the minds of employees. A good enterprise image can improve employees' satisfaction and identification with the company^40^. Liu et al.^41^ believe that enterprise image is the overall impression of an enterprise among the public. Based on the above research, it can be seen that enterprise image is the overall cognition of an enterprise among employees, which represents the degree of employees' identification with the enterprise. However, an enterprise image has a significant impact on employee loyalty and has a key role in employee retention^42^, and A positive enterprise image has a positive effect on employee loyalty^43^. Therefore, enterprise image may be positively correlated with employee satisfaction and loyalty. The following hypotheses are proposed:

### Hypothesis 2 (H2)

There is a significant positive correlation between enterprise image and employee satisfaction.

### Hypothesis 3 (H3)

There is a significant positive correlation between enterprise image and employee loyalty.

## Switching cost

Switching cost is an important reference factor for employees considering switching companies. When employees consider changing careers, they measure the costs and benefits of the change, and if the price of switching is higher than the benefit, i.e., if the switching cost is too high, exit barriers can be formed, making employee conversion less likely^44^. When employees' switching costs increase, their sensitivity to satisfaction will decrease^45^. A large number of studies have pointed out that the switching cost perceived by employees is an important factor in employee loyalty^44^. Dissatisfied employees may stay in their old companies simply because they would have to spend extra time and effort to change companies, which is costly. Therefore, the switching cost has a significant impact on employee satisfaction and loyalty. The following hypotheses are proposed:

### Hypothesis 4 (H4)

There is a significant positive correlation between switching cost and employee satisfaction.

### Hypothesis 5 (H5)

There is a significant positive correlation between switching cost and employee loyalty.

### Employee satisfaction and loyalty

In empirical studies, satisfaction is a prerequisite for loyalty^46^. However, these management drivers influence employee loyalty through satisfaction. In terms of the impact of management drivers, satisfaction is regarded as an endogenous variable, but in terms of the impact on employee loyalty, satisfaction is also an exogenous variable^16^. Therefore, according to the identification of the variable coefficient of the mediation variable^47^, satisfaction is considered the mediator of the model. Turkyilmaz et al.^28^ and Matzler et al.^48^ reported a significant mediating effect of employee satisfaction on employee loyalty. Employee job satisfaction has a positive impact on employee organizational loyalty^49^. According to Martensen and Gronholdt^50^, employee satisfaction is positively correlated with employee loyalty. In addition, a consistently strong relationship between organizational loyalty and the job satisfaction of employees has been reported^51^. Employee job satisfaction is positively correlated with loyalty. Therefore, the following hypothesis is proposed.

#### Hypothesis 6 (H6)

There is a significant positive correlation between employee satisfaction and employee loyalty.

**Figure 1:**
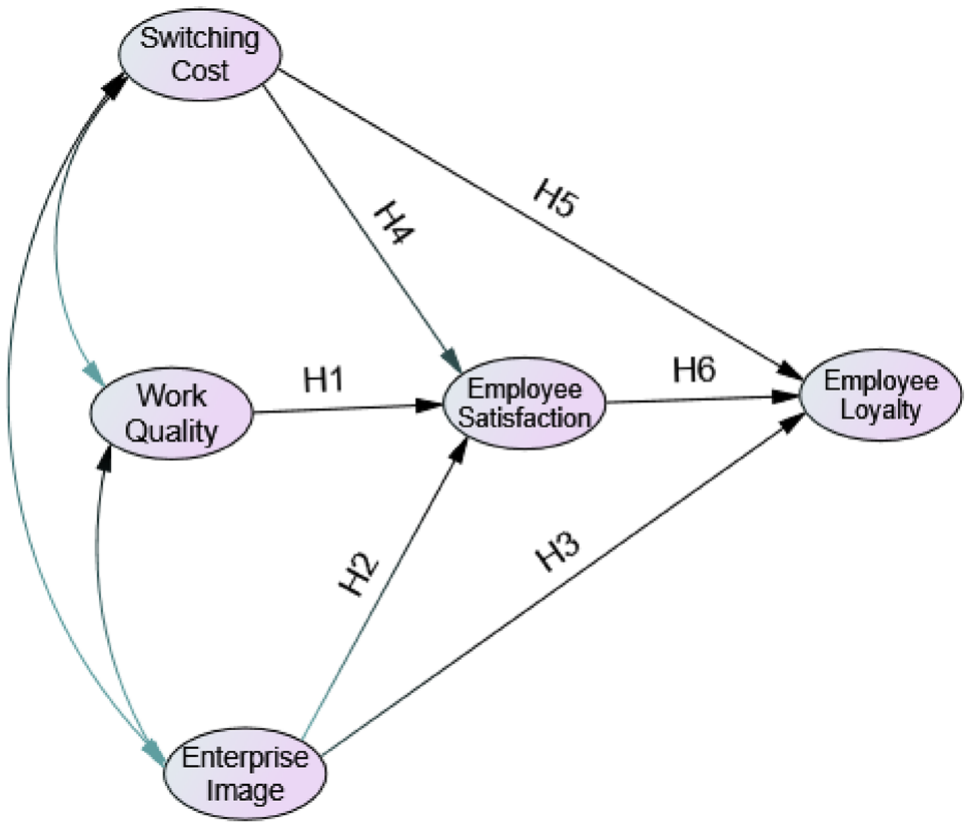
Conceptual model.

According to these hypotheses, a model was constructed, and the research framework developed in this study is shown in Fig. 1.

## Methodology

### Measurement and survey instrument

In the structural model of this study, all structural measurement items were adapted from the literature (see Table 1) and modified according to the feedback of three experts. The scale covers five dimensions of miners' loyalty. The dimension of loyalty scale is based on the work of Kumar and Shah^52–54^; the dimension of work quality scale is derived from Parasuraman^39^ and Hong et al.^55^; and the dimension of employee satisfaction scale was adapted from Ibanez et al.^39^, Liu et al.^41^, Anderson et al.^56^ and Zhang^57^. The measurement of enterprise image was adapted from the literature of Nguyen and LeBlanc^58^, Chang and Tu^42^ and Liu et al.^41^. Finally, the switching cost dimension scale was adapted from Jones et al.^44^, Fornell^59^ and Kim et al.^60^.

**Table 1:** Summary of items.

Variables	Code	Item content	References
Employee loyalty	EL1	I will mention the company's strengths to others	Kumar and Shah^52^, Lam et al.^53^, Zeithaml and Parasuraman^54^
	EL2	When I am asked to recommend a job, I recommend the company	
	EL3	I will actively recommend this company to my friends and family	
	EL4	I always consider what can be done to make progress in my current work	
	EL5	During my work, I shall not violate the relevant regulations of the enterprise	
	EL6	I am willing to use this company as my first choice for future work	
	EL7	I would like to continue working at this company in the future	
	EL8	I will continue to communicate with or engage in the company in the future	
	EL9	I would very much like to spend my entire career in my current company	
Work quality	Comfortability		Parasuraman^37^, Hong et al.^55^
	WQ1	The company's floors are clearly marked and easy to identify	
	WQ2	The company's music, decor and atmosphere are comfortable	
	WQ3	The company is conveniently located and easy to access or has available parking	
	WQ4	The company's facilities are well organized and well planned	
	WQ5	The staff of company are neatly dressed and well-groomed	
	Reliability		
	WQ6	The company operates an employee purchase insurance scheme	
	WQ7	The promises made by the company are faithfully fulfilled	
	WQ8	Jobs assigned by the company that are not suitable can be replaced quickly	
	WQ9	The requirements and workload of the company are consistent	
	Responsiveness		
	WQ10	The company's managers are happy to help their employees	
	WQ11	The leadership and management respond quickly to employees' requests	
	WQ12	Managers have the ability to solve employees’ problems	
	Assurance		
	WQ13	Proactively inform employees of the year-end performance bonus system	
	WQ14	I trust the information given to me by the management of the company	
	WQ15	I think the management is properly educated and trained	
	WQ16	The company's managers are very attentive to their employees	
	Empathy		
	WQ17	Individual requests for leave are granted in the case of a family emergency	
	WQ18	The company has a reasonable schedule of working hours	
	WQ19	The company's managers are aware of the needs of their employees	
	WQ20	The company will provide some allowance for food and accommodation	
	WQ21	I like that the company puts its employees first	
Employee satisfaction	ES1	I am satisfied with my current salary compared with others	Anderson et al.^55^, Ibanez et al.^39^, Zhang^57^, Liu et al.^41^
	ES2	I am satisfied with the company's welfare policy	
	ES3	I am satisfied with the company’s dormitory environment	
	ES4	I am satisfied with the position	
	ES5	I am satisfied with my job match	
	ES6	I am satisfied with the company's work safety and security	
	ES7	The company's systems reflect fairness and justice	
Enterprise image	EI1	The company has a high profile	Nguyen and LeBlanc^58^, Chang and Tu^42^, Liu et al.^57^
	EI2	The company's corporate identity is clear and easy to identify	
	EI3	The company occupies a leading position in the market	
	EI4	The company occupies a considerable place in my mind	
	EI5	The company actively participates in or sponsors social activities	
Switching cost	SC1	If I changed work, it would take me much time to reconnect with other people	Jones et al.^44^, Fornell^59^, Kim et al.^60^
	SC2	If I changed work, it would take me much time to readjust to the position	
	SC3	If I changed work, I would no longer enjoy the same privileges and benefits	
	SC4	If I changed work, I do not think the new company could offer the equivalent	
	SC5	I am used to this organization, so I do not want to change it	

Note: The Cronbach-a coefficients of employee loyalty, employee satisfaction, enterprise image, work quality and switching cost are 0.879, 0.780, 0.780, 0.835 and 0.821, respectively, and the total reliability of the questionnaire is 0.950.

To test the proposed model, a double translation protocol was utilized to develop the questionnaire^61^. The questionnaire was originally written in English and then translated into Chinese by three bilingual experts from a mine safety management school in China. Three bilingual Chinese mine management experts then translated the questionnaire from Chinese into English. There was no significant difference among the three English questionnaires^55^. Three experts were asked to review the statements and items in the questionnaire to make them clearly consistent with the hypothetical model^62^. The first expert is a full university professor who specializes in safety science in China and is a certified safety engineer and safety evaluator who has provided technical advice to a wide range of industries, including automobile manufacturers, mining and chemical industries. The other two experts are senior mine management engineers who have worked in the mining industry for many years and studied the safety management of miners' behavior. Based on the suggestions of the experts, we revised some questions to better suit the research context. In this study, the questionnaire was produced using the reverse translation strategy. In addition, this study adopted a seven-point Likert-type scale ranging from 1 (strongly disagree) to 7 (strongly agree) for all variables to measure the construct items^63^.

Prior to SEM analysis, the raw data were filtered to identify possible problems^47^. First, the missing values were excluded from the dataset. Second, the skewness index (SI) and kurtosis index (KI) were used to test the normality hypothesis of each index distribution. The SI reported in this study was between − 1.692 and − 0.699, and its absolute value was less than the recommended level of 3^47^. The KI values ranged from − 0.466 to 3.958, and the absolute value was less than 10^47^. Therefore, the normality assumption of these indicators was not rejected^16^.

### Participants and procedures

The research object of this paper is the miners of Pulang Copper Mine, which is the largest copper mine in China. It is located in southwest China at an altitude of 3500–4000 m, and the temperature is − 5 ~ − 20 °C. In this study, face-to-face questionnaires were employed. The data were collected over three weeks in the winter of 2019. Stratified random sampling was performed in this study according to the working characteristics of each miner and the number of miners in the whole mine. In total, 200 surface miners, 250 underground miners and 50 surface managers were selected as the survey samples. Hence, a total of 500 questionnaires were distributed during the period, and 478 questionnaires were collected with a recovery rate of 95.6%. After the elimination of incomplete and invalid questionnaires, 440 useful responses were obtained for data analysis, with an effective rate of 92.1%. Among the respondents returning valid questionnaires, the sample was mostly male (91.3%) and mostly 30–39 years old (68.5%). Most of the respondents had 5–10 years (78.6%) of working experience in their current company.

### Ethical approval and consent to participate

This study was approved by the Research Ethics Committee of Northeastern University (23-2019-0105). All methods were carried out in accordance with relevant guidelines and regulations. Informed consent was obtained from all subjects following a detailed explanation of the study objectives and protocol to each subject. All subjects provided written informed consent prior to being monitored.

### Data analysis

#### Statistical analysis (data processing)

©IBM SPSS 22.0 and ©IBM SPSS AMOS 24.0 were used for statistical analyses in this paper. Basic descriptive analysis was performed to obtain scores for the five dimensions of the questionnaire, and descriptive statistics (mean, standard deviation, standard error and confidence interval [CI = 95%]) were calculated^64^. Pearson correlation analysis was used to verify the correlations between study variables. The relationships between demographic factors, factors related to miners' satisfaction and loyalty factors were used to predict miners' psychological status, and path analysis (SEM-maximum likelihood estimation) was used to test the model with a significance level of *p* < 0.05, *p* < 0.01 and *p* < 0.001. SEM is a multivariable technique that follows a conceptual model, path analysis diagram and relational regression equation system^65^. The SEM is a comprehensive statistical method for testing hypotheses about the relationship between observed variables and potential variables. The SEM combines the characteristics of factor analysis and multiple regression to analyze the measure and structure of a theoretical model. The SEM is formally defined by two sets of linear equations: inner model and outer model. The inner model specifies the relationship between two unobserved or potential variables, while the outer model specifies the relationship between the potential variables and their associated observed or display variables^66^.

#### Reliability and validity analysis

In this paper, reliability and validity tests were used to examine the five latent variables and their constructs. First, the reliability of each construct was tested. Reliability represents the variance of a measurement resulting from repeated measurements of the same concept. It is related to nonsystematic errors and can be expressed as stability, consistency, predictability and accuracy^54^. Cronbach’s alpha was used for the reliability test. Cronbach's alpha is a measure of the reliability of a scale or test that overcomes the disadvantage of partial splitting. Cronbach’s alpha is the most commonly employed reliability analysis method in social science research. In basic research, the reliability measure coefficients of all components exceeded the threshold value of 0.70^67^, and each measurement remained above the values of 3 explanatory variables to achieve a correctly identified or overidentified model. Validity refers to the accuracy of the measurement. The purpose of principal component analysis is to find the most meaningful basis and express the similarities and differences in the data. In addition, confirmatory factor analysis (CFA) is a way to test how well a variable represents the construct. CFA verification results can provide evidence for the convergence and discriminant validity of theoretical constructs^68^. In this paper, the higher-order mediation model is involved. Previous studies have shown that the advantage of using the second-order factor model in SEM is mainly to simplify the model and program for easy interpretation^69^. The target coefficient (first-order measurement model χ^2^/second-order measurement model χ^2^) is a method to measure the second-order model. The target coefficient (T) is close to one, indicating that the second-order model is more representative. However, when T is 0.74, it is acceptable^70^. For example, work quality in this paper is a multidimensional structure with second-order latent variables in four structures: comfortability, reliability, responsiveness and empathy. To measure second-order factors, first-order factors are used as dependent variables^71^. Regarding the square root of the latent variable average variance extraction (AVE), to obtain sufficient discriminant validity, the square root of any potential variable’s AVE should be greater than the correlation between it and other potential variables^72^.

Reliability is an indicator of convergent validity, and we assessed the latent factors’ reliability by calculating a composite reliability for each construct^73^. This value can be calculated using standardized loadings:

1 $$CR = \frac{{\left( {\sum\nolimits_{i = 1}^{n} {L_{i} } } \right)^{2} }}{{\left( {\sum\nolimits_{i = 1}^{n} {L_{i} } } \right)^{2} + \left( {\sum\nolimits_{i = 1}^{n} {e_{i} } } \right)}}$$

where $CR$ is the composite reliability for the scale, $L_{i}$ represents the standardized factor loading and $e_{i}$ is the measurement error for scale items. In addition to the reliability calculations, we examined the parameter estimates and their associated t-values as well as the average variances extracted^73^. The average variance extracted was calculated as follows:

2 $$AVE = \frac{{\sum\nolimits_{i = 1}^{n} {L_{i}^{2} } }}{n}$$

where $AVE$ is the average variance extracted and $L_{i}$ represents the standardized factor loading.

#### Fit indices

Model fitting can be divided into three categories: absolute fitting (Chi-square (χ^2^), root mean square error approximation (RMSEA) and goodness-of-fit index (GFI)), incremental fitting (adjusted goodness-of-fit index (AGFI), comparative fit index (CFI), Tucker- Lewis index (TLI), normed fit index (NFI)), and parsimonious fitting (Chi-square/degree of freedom (df))^72^. Holms-Smith et al.^74^ and Hair et al.^75^ suggest using at least one fitness index for each model fit category. Chi-square/df should fall between 1 and 5; the CFI should be greater than 0.90; the standardized root mean square residual (SRMR) should be less than 0.08; and the RMSEA should be less than 0.06^76^. Chi-square/df, GFI, AGFI, CFI and RMSEA were used as the measurements of model fitting in the analysis of this study.

## Data analysis and results

The model was analyzed using IBM SPSS 22.0 and IBM SPSS AMOS 24.0 software. First, descriptive statistics were carried out on the test items; there was no multicollinearity problem or common method variability between the items, proving that there were no offending estimations. Second, the measurement model of CFA directly influences the quality of SEM, and SEM is almost the mean of CFA, indicating that the model is suitable for this study. Finally, the causal effect of the structural model is analyzed by using the parameters of the SEM.

### Descriptive statistics

Table 2 shows that all variables were significantly correlated (*p* < 0.01). However, the correlation values did not exceed the cutoff of 0.90, indicating that there was no multicollinearity problem between items^77^. The study adopted a subjective survey for measurement. However, the results may present the risk of common method variance^16^. Hence, we also used Harman's single-factor test to detect common method bias in the data^78^. By adding all the observations together and analyzing factors or components, the cumulative percentage of explanatory variation under the unrotated axis was 51.946%^79,80^. Therefore, common method bias was not evident.

**Table 2:** Descriptive statistics and correlations among variables.

Construct scales	Mean	SD	Employee loyalty	Work quality	Employee satisfaction	Enterprise image	Switching cost
Employee Loyalty	5.751	0.923	1				
Work Quality	5.326	1.088	0.704**	1			
Employee Satisfaction	5.270	1.193	0.628**	0.856**	1		
Enterprise Image	5.541	1.041	0.689**	0.781**	0.797**	1	
Switching Cost	5.290	1.190	0.599**	0.762**	0.767**	0.734**	1

**Correlation is significant at the 0.01 level, N = 440.

### Measurement model

**Figure 2:**
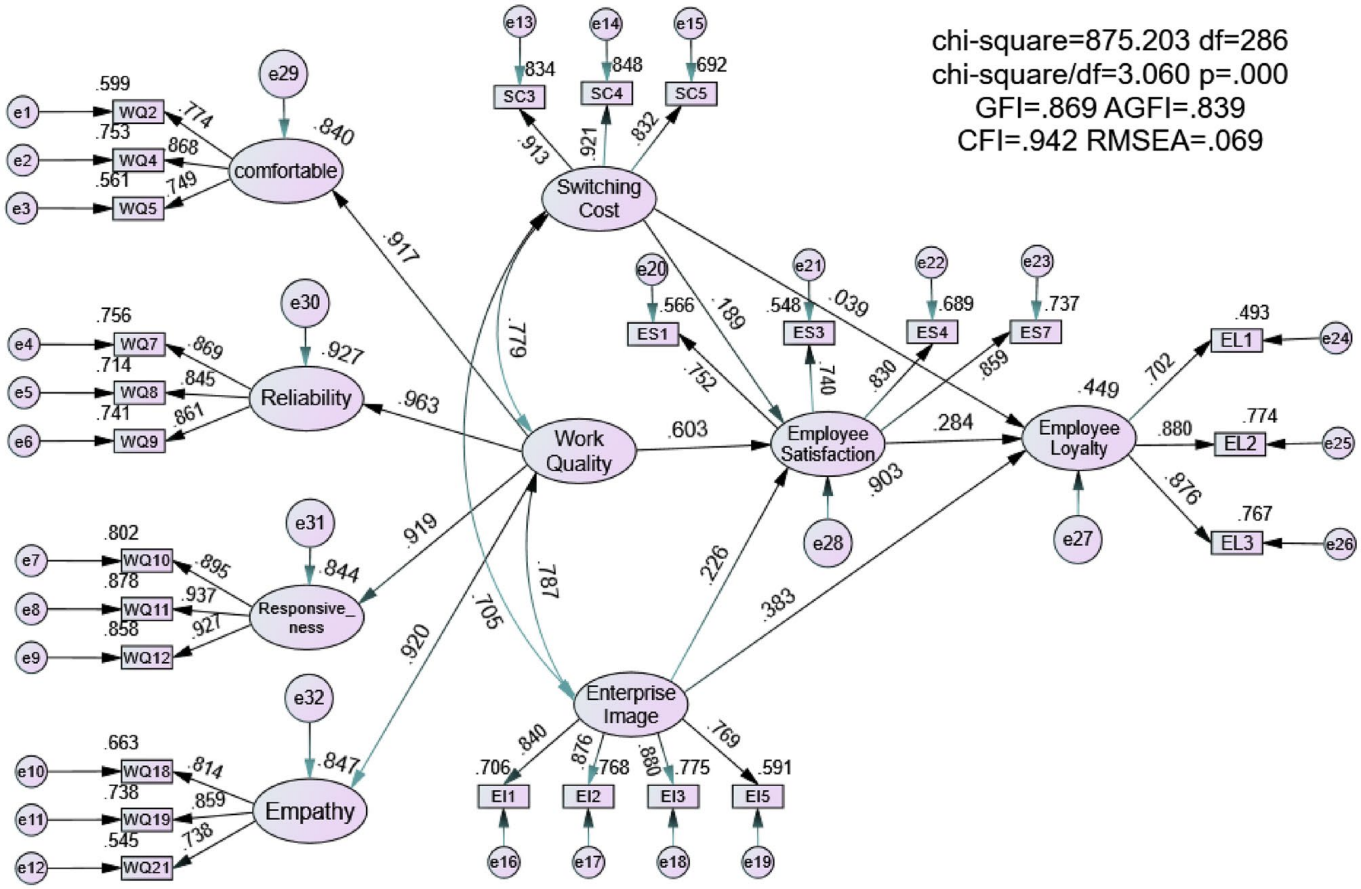
Structural equation model showing standardized path coefficients.

CFA plays a key role in measurement model verification in path or structure analysis^68,81^. When conducting SEM analysis, researchers usually evaluate the measurement model before discussing possible structural models. The measured variables accurately reflect the structure or factors. In general, problems with SEM are caused by measurement model problems that can be identified by CFA^68^. Therefore, AMOS was used for CFA, covariance matrix and maximum likelihood estimation. The results of the model include five factors and a total of 36 indicators. In the CFA measurement model shown in Fig. 2, items with standardized factor loads less than 0.6 are considered for deletion^82^. However, as shown in Table 3, the measurement model achieves a good fit, and all indicators related to potential factors show significant differences (*P* < 0.001).

**Table 3:** The convergent validity of factors.

Constructs	Items	Parameter significance estimation				Factor loading	Item reliability	Composite reliability	Convergent validity
		UnStd	S.E	T-value	*P*	Std	SMC	CR	AVE
Responsive-ness	WQ10	1.000				0.885	0.783	0.942	0.845
	WQ11	1.124	0.036	31.085	***	0.949	0.901		
	WQ12	1.027	0.035	29.603	***	0.922	0.850		
Reliability	WQ7	1.000				0.862	0.743	0.894	0.737
	WQ8	1.096	0.052	21.230	***	0.852	0.726		
	WQ9	0.933	0.044	21.446	***	0.861	0.741		
Empathy	WQ18	1.000				0.830	0.689	0.846	0.648
	WQ19	1.080	0.064	16.750	***	0.852	0.726		
	WQ21	0.888	0.058	15.430	***	0.728	0.530		
Comfortability	WQ2	1.000				0.720	0.518	0.840	0.639
	WQ4	1.144	0.077	14.817	***	0.916	0.839		
	WQ5	0.842	0.057	14.667	***	0.749	0.561		
Enterprise image	EI1	1.000				0.847	0.717	0.906	0.708
	EI2	1.103	0.047	23.716	***	0.897	0.805		
	EI3	1.103	0.048	22.813	***	0.872	0.760		
	EI5	0.940	0.053	17.837	***	0.740	0.548		
Employee satisfaction	ES1	1.000				0.774	0.599	0.878	0.644
	ES3	0.900	0.056	16.106	***	0.757	0.573		
	ES4	1.040	0.056	18.582	***	0.876	0.767		
	ES7	1.039	0.061	17.058	***	0.797	0.635		
Switching cost	SC3	1.000				0.924	0.854	0.918	0.789
	SC4	1.023	0.035	28.906	***	0.922	0.850		
	SC5	0.891	0.038	23.450	***	0.815	0.664		
Employee loyalty	EL1	1.000				0.686	0.471	0.861	0.677
	EL2	1.376	0.089	15.514	***	0.915	0.837		
	EL3	1.381	0.088	15.614	***	0.851	0.724		
Work quality	Co	1.000				0.779	0.607	0.878	0.642
	Re	1.315	0.071	18.462	***	0.866	0.750		
	Res	1.403	0.084	16.614	***	0.777	0.604		
	Emp	1.503	0.090	16.692	***	0.780	0.608		

***Correlation is significant at the 0.001 level.

Next, the convergent validity and discriminant validity of the measurement model factors were tested. Convergent validity refers to the aggregation or sharing of a high proportion of variance in a certain dimension^75^. Convergent validity can be estimated by factor loading and AVE. Discriminant validity refers to the degree of difference and evidence of excessive correlation between a factor and other constructs^75^. In the first-order factor model, the AVE is used to measure the degree of difference between each construct and other constructs. The mean variance of each structure is shown in Table 4, and the relationship between the variance caused by relevant basic factors and the variance caused by measurement error is measured. All values are higher than the minimum of 0.5 recommended by Fornell and Larcker^83^. In addition, the square root of AVE is greater than the maximum correlation between constructs (the shared variance between the two constructs), indicating significant discriminant validity^13^. As shown in Table 4, the five factors do not have convergent and discriminant validity or reliability problems in this study.

**Table 4:** The results of discriminant validity.

Construct	AVE	Work quality	Employee loyalty	Employee satisfaction	Enterprise image	Switching cost
Work quality	0.642	**0.801**				
Employee loyalty	0.677	0.642	0.**823**			
Employee satisfaction	0.644	0.925	0.608	**0.802**		
Enterprise image	0.708	0.785	0.646	0.834	**0.841**	
Switching cost	0.789	0.779	0.540	0.817	0.705	**0.888**

Diagonals (in bold) represent the square root of the average variance extracted, while the other entries represent the correlations.

Specifically, the first order and the second order were analyzed and tested with CFA. Marsh and Hocevar^84^ determined the fitness of the data by calculating the target coefficient (T) and comparing first-order and second-order CFA. A T value close to one indicates that second-order CFA can replace first-order CFA, making the model more precise and simplified^85^. The T value of work quality in this study is 0.940 (see Table 5), which is close to the target coefficient. The fitness index of the second-order CFA of work quality shows that its fitness is good and simplified. Therefore, this study uses the results of second-order CFA to implement SEM analyses.

**Table 5:** The goodness-of-fit indexes for work quality, first-order and second-order factor models.

First—and second-order factor models	χ^2^	df	χ^2^/df	GFI	AGFI	CFI	RMSEA
0. Null model	4641.403	66	70.324	0.182	0.034	0	0.397
1. First-order one-factor model	430.478	54	7.972	0.845	0.776	0.918	0.126
2. First-order four-factor model (no correlation between factors)	1547.943	54	28.666	0.621	0.453	0.673	0.251
3. First-order four-factor model (correlation between factors)	124.277	48	2.589	0.955	0.928	0.983	0.060
4. Second-order factor model	132.101	50	2.642	0.953	0.927	0.982	0.061
5. Target coefficient (T)	0.940						

*GFI* goodness-of-fit index, *AGFI* adjusted GFI, *CFI* comparative fit index, *RMSEA* root mean square error approximation. Target coefficient (T) = Chi-square value for first-order four-factor model (factors are correlated)/chi-square value for second-order four-factor model;

χ^2^ test = Chi-square test.

### Structural model assessment

According to the research hypothesis, the model was tested through SEM analysis (as shown in Fig. 2). Figure 2 presents the standardized path coefficient estimation model. The fitness index of the structural model was χ^2^ = 875.203 (*P* = 0.000), df = 286, χ^2^/df = 3.060, GFI = 0.869, AGFI = 0.839, CFI = 0.942, RMSEA = 0.069. Comparison of the results with the corresponding critical values showed that the conceptual model fit the empirical data well^86^. The critical values of model fitting are as follows: χ^2^/df below 5 is acceptable, GFI and AGFI are > 0.8, CFI value is > 0.9, and RMSEA is < 0.08 (less than 0.05 is better). Therefore, the model has satisfactory explanatory ability and robustness, as shown in Table 3 and represented graphically in Fig. 2. All standardized parameter estimates are shown in Fig. 2, in which the unidirectional arrows represent the direction of the predictive relationship and bidirectional arrows indicate the correlation between two study variables^87^.

**Figure 3:**
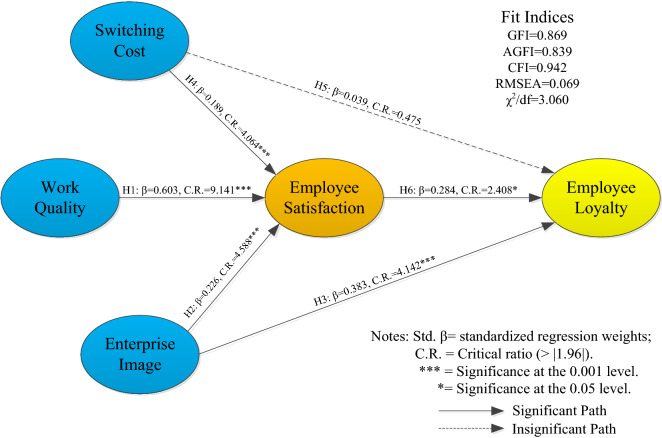
Results of structural equation modeling.

The hypothesis testing results (refer to Fig. 3 and Table 6) show that the influence of enterprise image on employee loyalty is significantly positive (standardized β = 0.383, C.R. = 4.142), indicating that the stronger the influence between enterprise image and society is, the more likely employees are to be loyal to the company. Therefore, H3 is confirmed. The influence of employee satisfaction on employee loyalty is significantly positive (standardized β = 0.284, C.R. = 2.408), indicating that when employees are satisfied with their work, they demonstrate greater loyalty to the enterprise, that is, employee satisfaction has a positive impact on employee loyalty. Thus, H6 is confirmed. Regarding the direct effects of three characteristic predictors on employee satisfaction (standardized β = 0.603, C.R. = 9.141; β = 0.226, C.R. = 4.588; β = 0.189, C.R. = 4.064), all paths showed significant direct effects, supporting H1, H2, and H4. In terms of the mediating effect of employee satisfaction, the nonsignificant coefficient of the direct influence between switching cost and employee loyalty (standardized β = 0.039, C.R. = 0.475(< 1.96)) indicated a causal relationship between switching costs and employee loyalty through the complete mediating effect of employee satisfaction. Moreover, work quality and enterprise image have a significant impact on employee loyalty via their connection between employee satisfaction and employee loyalty. Therefore, hypothesis H5 is rejected, while H1, H2 and H4 are supported. The three predictors of the explicit variables represent the three aspects of conversion cost, work quality and enterprise image, all of which have a significant influence on employee loyalty through all or part of the mediating effect generated by satisfaction. Table 7 presents these influences (i.e., direct, indirect and total effects). However, both the indirect effect and the direct effect of enterprise image on employee loyalty through employee satisfaction were significant (β = 0.064 and β = 0.383, respectively) (refer to Fig. 3 and Table 7). The results confirmed that employee satisfaction partially mediates the relationship between enterprise image and employee loyalty. The indirect effect of work quality and switching cost on employee loyalty through employee satisfaction was significant, whereas the corresponding direct effect was nonsignificant (refer to Fig. 3 and Table 7). The results confirmed that employee satisfaction fully mediates the relationship between work quality and switching cost and employee loyalty.

**Table 6:** Results of the significance test of the model and hypothesis testing.

Hypothesis/path	Estimate	S.E	C.R	*P*	Std. estimate (β)	Hypothesis testing results
H1. Work quality → Employee satisfaction	0.644	0.070	9.141	***	0.603	Supported H1
H2. Enterprise image → Employee satisfaction	0.279	0.061	4.588	***	0.226	Supported H2
H3. Enterprise image → Employee loyalty	0.328	0.079	4.142	***	0.383	Supported H3
H4. Switching cost → Employee satisfaction	0.166	0.041	4.064	***	0.189	Supported H4
H5. Switching cost → Employee loyalty	0.024	0.050	0.475	0.635	0.039	Rejection H5
H6. Employee satisfaction → Employee loyalty	0.196	0.081	2.408	0.016	0.284	Supported H6

Estimate = Unstandardized regression weights; Std. Estimate (β) = standardized regression weights; S.E. = Standardized error; C.R. = Critical ratio ( >|1.96|). *** = Significance at the 0.001 level.

**Table 7:** Direct, indirect, and total effects of employee satisfaction and employee loyalty.

Path	Direct effect	Indirect effect	Total effect
Work quality → Employee satisfaction	0.603	–	0.603
Enterprise image → Employee satisfaction	0.226	–	0.226
Switching cost → Employee satisfaction	0.189	–	0.189
Employee satisfaction → Employee loyalty	0.284	–	0.284
Enterprise image → Employee loyalty	0.383	0.064	0.447
Switching cost → Employee loyalty	0.039	0.054	0.093
Work quality → Employee loyalty	–	0.171	0.171

The total effect of one construct on another is the sum of the direct effect and indirect relationships between them. The indirect effect is computed by multiplying the direct effects by each other, e.g., the indirect effect of Work Quality → Employee Loyalty is computed as 0.603 × 0.284 = 0.171.

## Discussion

Currently, with the rapid economic development in the industrial environment, employees expect enterprises to quickly provide safe, comfortable and healthy working conditions and satisfactory remuneration, which enterprises tend to ignore in reality in order to obtain greater benefits. However, the contradiction between safety and production is the root cause of safety accidents. Therefore, controlling for the employee's dynamic psychological characteristics (employee satisfaction, loyalty) and adjusting the system of safety management measures and psychological characteristics to adapt to the characteristics of the psychological contract, the safety and risk awareness of staff can be improved, errors can be reduced, personnel can be screened, and the occurrence of safety accidents can be curbed. This kind of security management thinking becomes particularly important. Therefore, the importance of the dynamic perception of employees' psychological status is widely recognized in most fields and a focus of enterprise institutional decision-making, especially in high-risk production industries.

This paper empirically studies employee satisfaction and loyalty in a high-risk industry in an extreme environment. To clarify these relationships, a research model was proposed to analyze the relationships among five constructs. The model has six hypotheses, which were tested by using data collected from miners in China's largest metal mining company. The reliability and robustness of the model are verified, and the results are satisfactory.

The results show that enterprise image (H2: β = 0.226) and switching costs (H4: β = 0.189) have a positive impact on employee satisfaction (refer to Fig. 3 and Table 6). This result suggests that improving enterprise image and reputation and employee treatment are important to employee satisfaction^88^. This finding is similar to those of previous studies^89,90^. Clearly, enterprise image and employee treatment are very important to improve employee loyalty and material satisfaction. Moreover, the enterprise image (H3: β = 0.383) has positive direct effects on employee loyalty by providing employees information regarding the recognition of the enterprise. Interestingly, the field investigation showed that switching costs (H5: β = 0.039) have no significant impact on employees' degree of loyalty because the mining enterprise is located in a remote area with few alternative employment options, which greatly limits employees’ career prospects. Therefore, employees are mostly forced to work at this company because the decision to resign and choose a new enterprise would be costly. The employees’ psychological characteristics lead them to continue working at the high-risk enterprise.

In particular, this study also found a positive correlation between work quality and employee satisfaction (H1: β = 0.603), and the effect was very significant. Moreover, employee loyalty was positively correlated with work quality (H1: β = 0.603) and employee satisfaction (H6: β = 0.284) (refer to Figs. 2, 3). This result makes sense because the work quality is based on the comfort of the work environment, staff requirements and the reliability of work safety, the responsiveness of enterprise management to the staff and an enterprise management system with a focus on employees. These four characteristics indicate that the quality of work is superior, reflecting employee satisfaction and, in turn, employee loyalty^91,92^. Therefore, enterprise managers should create a good working environment and humanized management to improve employees' safety behavior and loyal attitude.

The research results are of great significance to mine safety management, especially for reducing unsafe behaviors on site and highlighting employees' dynamic psychological changes. Psychological contract theory and employee satisfaction and loyalty are applied in the context of safety behavior, and the key role of employee psychology in safety management is realized, which provides a new direction for mine safety research. Through the real-time investigation and study of this loyalty model, an enterprise can identify the psychological changes of short-term employees, grasp which safety systems and decision-making regulatory policies of the enterprise do not fully meet the requirements of workers, and carry out real-time improvement and optimization of safety systems.

Academically, this study contributes in several ways. This study contributes to the empirical testing of the impact of work quality, enterprise image and switching costs on employee satisfaction and employee loyalty in extremely high-altitude and low-oxygen environments. Moreover, it seeks new safety management methods to effectively and safely improve production performance and enhance employee satisfaction and loyalty by improving work quality, for example, by optimizing the production process system, improving the treatment of employees to prevent brain drain, shaping a good enterprise image, improving work conditions, and improving employee satisfaction and loyalty. According to the questionnaire survey of miners' cognition of working environment conditions, enterprise image and their psychological dynamics of job satisfaction and loyalty, dynamic monitoring of miners' psychological changes during practical production was performed through this research model. The enterprise management mode and system were adjusted in real time. In addition, the model of this study is helpful for the practice of dynamic, real-time monitoring of mining employees' psychological dynamics as a reference for enterprise decision makers in safety management.

This study has some limitations. A large amount of data were collected through questionnaires, and opinions were solicited from department leaders and front-line workers. The questionnaire survey, which was conducted in only one mine in one region, was not compared across multiple situations from a large diversity of locations and environments to reduce the chance that the results would be valid for only a single case. However, the models and data obtained are not universally applicable. Considering that a large number of employees participated in the data collection and that the level of knowledge varies greatly across employees, the interviewees may have offered vague judgments on some investigation factors. Therefore, it may not be possible to completely control the implementation of the questionnaire survey. In addition, this study mainly investigated the employees of mining enterprises in high-altitude and cold areas to establish an employee loyalty model. Hence, the universality of this research model may be limited.

Future research should account for the above limitations. In the future, we plan to increase the data collection volume and expand the scope of the survey area. Moreover, we will extend the model to other employees through further survey research to improve the interpretation ability and general adaptability of the model. Another direction for future research would be to develop a more comprehensive computer-based sample collection technique to analyze the psychological security factors of employee loyalty in different industries or areas.

## Conclusion

This study quantitatively analyzed employee satisfaction and factors that affect employee loyalty in the high-risk mining industry in China. The findings of the study demonstrated the important characteristics of satisfaction as a driving factor and mediating variable with direct and indirect relationships with mine workers' loyalty. The results show that there is a linear positive correlation between employee satisfaction and employee loyalty in mining enterprises. Specifically, employee loyalty is significantly related to enterprise image and employee satisfaction. Work quality indirectly affects employee loyalty through satisfaction, while switching costs have no significant impact on employee loyalty. Accurate dynamic and real-time monitoring of the psychological dynamics of the employee satisfaction level and loyalty of workers in high-risk industries is very important for improving work quality and safety. We provide strong empirical evidence and conceptual models that can play an important role in improving sustainable HRM and operational performance as well as institutional decision-making regarding safety.
